# Learning, Memory, and the Role of Neural Network Architecture

**DOI:** 10.1371/journal.pcbi.1002063

**Published:** 2011-06-30

**Authors:** Ann M. Hermundstad, Kevin S. Brown, Danielle S. Bassett, Jean M. Carlson

**Affiliations:** Physics Department, University of California, Santa Barbara, Santa Barbara, California, United States of America; Indiana University, United States of America

## Abstract

The performance of information processing systems, from artificial neural networks to natural neuronal ensembles, depends heavily on the underlying system architecture. In this study, we compare the performance of parallel and layered network architectures during sequential tasks that require both acquisition and retention of information, thereby identifying tradeoffs between learning and memory processes. During the task of supervised, sequential function approximation, networks produce and adapt representations of external information. Performance is evaluated by statistically analyzing the error in these representations while varying the initial network state, the structure of the external information, and the time given to learn the information. We link performance to complexity in network architecture by characterizing local error landscape curvature. We find that variations in error landscape structure give rise to tradeoffs in performance; these include the ability of the network to maximize accuracy versus minimize inaccuracy and produce specific versus generalizable representations of information. Parallel networks generate smooth error landscapes with deep, narrow minima, enabling them to find highly specific representations given sufficient time. While accurate, however, these representations are difficult to generalize. In contrast, layered networks generate rough error landscapes with a variety of local minima, allowing them to quickly find coarse representations. Although less accurate, these representations are easily adaptable. The presence of measurable performance tradeoffs in both layered and parallel networks has implications for understanding the behavior of a wide variety of natural and artificial learning systems.

## Author Summary

Information processing systems, such as natural biological networks and artificial computational networks, exhibit a strong interdependence between structural organization and functional performance. However, the extent to which variations in structure impact performance is not well understood, particularly in systems whose functionality must be simultaneously flexible and stable. By statistically analyzing the behavior of network systems during flexible learning and stable memory processes, we quantify the impact of structural variations on the ability of the network to learn, modify, and retain representations of information. Across a range of architectures drawn from both natural and artificial systems, we show that these networks face tradeoffs between the ability to learn and retain information, and the observed behavior varies depending on the initial network state and the time given to process information. Furthermore, we analyze the difficulty with which different network architectures produce accurate versus generalizable representations of information, thereby identifying the structural mechanisms that give rise to functional tradeoffs between learning and memory.

## Introduction

Learning, the assimilation of new information, and memory, the retention of old information, are competing processes; the first requires flexibility and the second stability in the presence of external stimuli. Varying structural complexity could uncover tradeoffs between flexibility and stability, particularly when comparing the functional performance of structurally distinct learning systems. We use neural networks as model learning systems to explore these tradeoffs in system architectures inspired by both biology and computer science, considering layered structures like those found in cortical lamina [1] and parallel structures such as those used for clustering [2], image processing [3], and forecasting [4]. We find inherent tradeoffs in network performance, most notably between acquisition versus retention of information and between the ability of the network to maximize success versus minimize failure during sequential learning and memory tasks. Identifying tradeoffs in performance that arise from complexity in architecture is crucial for understanding the relationship between structure and function in both natural and artificial learning systems.

Natural neuronal systems display a complex combination of serial and parallel [5] structural motifs which enable the performance of disparate functions [6]–[9]. For example, layered [1] and hierarchical [10] architectures theoretically important for sustained limited activity [11] have been consistently identified over a range of spatial scales in primate cortical systems [12]. Neurons themselves are organized into layers, or “lamina,” and both intra-laminar [13] and inter-laminar [14] connectivity differentially impact function. Similarly, information processing systems developed by technological innovation rather than natural evolution have structures designed to match their functionality. For example, the topological complexity of very large integrated circuits scales with the function to be performed [15]. Likewise, the internal structure of artificial neural networks can be carefully constructed [16] to enable these systems to learn a variety of complex relationships. While parallel, rather than serial, structures are appealing in artificial neural networks because of their efficiency and speed, variations in structure may provide additional benefits or drawbacks during the performance of sequential tasks.

The dependence of functional performance on structural architecture can be systematically examined within the framework of neural networks, where the complexity of both the network architecture and the external information can be precisely varied. In this study, we evaluate the representations of information produced by feedforward neural networks during supervised, sequential tasks that require both acquisition and retention of information. Our approach is quite different from studies in which large, dense networks are given an extended period of time to produce highly accurate representations of information (e.g. [17], [18]). Instead, we investigate the links between structure and function by performing a statistical analysis of the error in the representations produced by small networks during short training sessions, thereby identifying mechanisms that underlie tradeoffs in performance. Our work therefore has important implications for understanding the behavior of larger, more complicated systems in which statistical studies of performance would be impossible.

In the remainder of the paper, we discuss the extent to which network architectures differ in their ability to both learn and retain information. We first describe the network model and architectures considered in this study. We then quantify the best, worst, and average performance achieved by each network during sequential tasks that vary in both their duration and complexity. We consider the adaptability of these networks to variable initial states, thereby probing the structure of functional error landscapes. Finally, we explore how landscape variations that arise from structural complexity lead to differences in performance.

## Models

### Sequential Learning Approach

Our approach differs from traditional machine learning studies in that our goal is not to design the optimal network system for performing a specific task. Rather, we identify tradeoffs in network performance across a range of architectures that share a common algorithmic framework. In this context, the term “architecture” refers specifically to the structural organization of network connections and not, as is found in engineering studies, to the broader set of constraints governing the interactions of network components.

In evaluating network performance, we use techniques relevant to both artificial and biological systems. Artificial network systems often favor high accuracy and consistency during a single task, regardless of the time required to achieve such a solution. In biological systems, however, speed and generalizability are often more important that absolute accuracy when dynamically adapting to a variety of tasks. To probe features such as network accuracy, consistency, speed, and adaptability, we examine the representations of information produced by neural networks during competing learning and memory tasks.

We choose to study learning and memory within the biologically-motivated framework of feedforward, backpropagation (FFBP) artificial neural networks that perform the task of supervised, one-dimensional function approximation. The training process, which consists of adjusting internal connection strengths to minimize the network error on a set of external data points, can be mapped to motion within a continuous error landscape. Within this context, “learning” refers to the ability of the network to successfully navigate this landscape and produce an accurate functional representation of a set of data points, while “memory” refers to the ability to store a representation of previously-learned information. Additional details of this framework are described in the following subsection.

To simultaneously study learning and memory processes, information must be presented to the network sequentially. “Catastrophic forgetting,” in which a network learns new information at the cost of forgetting old information, is a longstanding problem in sequential training of neural networks and has been addressed with several types of rehearsal methods [19]–[21]. Standard rehearsal involves training the network with both the original and new information during sequential training sessions. We use a more biologically motivated approach, the pseudorehearsal method [22], in which the network trains with a *representation* of the original information. Pseudorehearsal has been shown to prevent catastrophic forgetting in both feedforward and recurrent networks and does not require extensive storage of examples [22], [23].

In training FFBP networks, local minima and plateaus within the error landscape can prevent the network from finding a global optimum [24], [25]. While considered disadvantageous in machine learning studies, the existence of local minima may provide benefits during the training process, particularly in biological systems for which highly accurate global optimums may be unnecessary or undesirable. Additionally, FFBP networks can suffer from overfitting, a problem in which the creation of highly specific representations of information hinders the ability of the network to generalize to new situations [26]. While also considered disadvantageous, failure to generalize has important biological consequences and has been linked to neurological development disorders such as Autism [27]. Instead of attempting to eliminate these sensitivities, we seek to understand the architectural basis for differences in landscape features and examine their impact on representational capabilities such as specificity and generalizability.

### Neural Network Model

**Figure 1:**
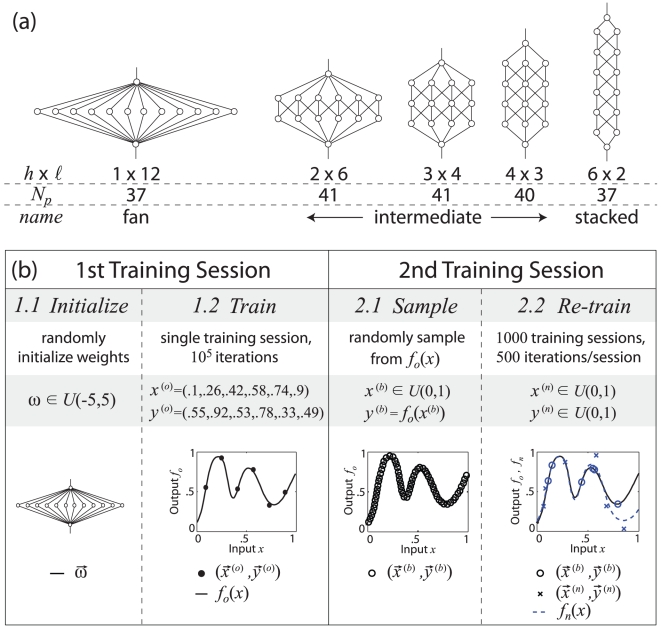
Network architectures and training task. (a) Network architectures considered in this study. Indicated below each network are the number of hidden layers 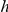 and nodes per layer 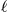, the total number of adjustable parameters 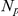, and the name by which we refer to the network. (b) Illustration of the sequential learning task described in the text applied to the fan network. Each step of the task includes a concise description of the procedure and the choice of network weights and training data.

The construction of our network model is consistent with standard FFBP neural network models [26]. We consider the five distinct architectures shown in Figure 1(a), all of which obey identical training rules. Each network has 12 hidden nodes arranged into 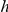 layers of 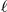 nodes per layer. Nodes in adjacent layers are connected via variable, unidirectional weights. The “fan” and “stacked” networks are both fully connected and have the same total number of connections. The connectivities of the “intermediate” networks, which have slightly greater numbers of connections, were chosen in order to roughly maintain the same total number of adjustable parameters per network, 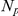, noted in Figure 1(a).

Each node has a sigmoid transfer function 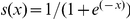 with a variable threshold 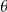. The output 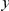 of each node is a function of the weighted sum of its inputs 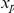, given by 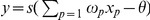, where 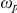 gives the weight of the 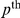 input connection. Representing the threshold as 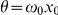, where 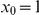 for all nodes, allows us to organize all adjustable parameters into a single, 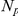-dimensional weight vector 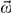.

During training, each network is presented with a training pattern of 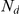 pairs of input 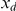 and target 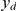 values, denoted 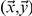. We restrict the input 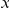 space to the range 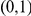, and the sigmoid transfer function restricts the output 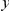 space to the range 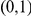. The set of variable weights 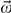 is iteratively updated via the Polak-Ribiere conjugate gradient descent method with an adaptive step size [28]–[30] in order to minimize the output error 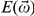. We use online training, for which 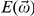 is the sum of squared errors between the network output 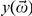 and target output 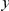 calculated after all 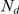 points are presented to the network:
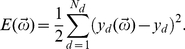
(1)


### Task Implementation

Each network shown in Figure 1(a) is trained over two sequential sessions. In describing parameter choices for each training session, we use 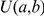 to denote a continuous uniform probability distribution over the interval 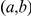. The steps of the sequential training process are shown schematically in Figure 1(b) and are described below:

### First Training Session

#### Step 1.1 - Initialize

Network weights are randomly chosen from 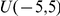. We refer to this state of the network as the “randomly initialized state”.

#### Step 1.2 - Train

**Figure 2:**
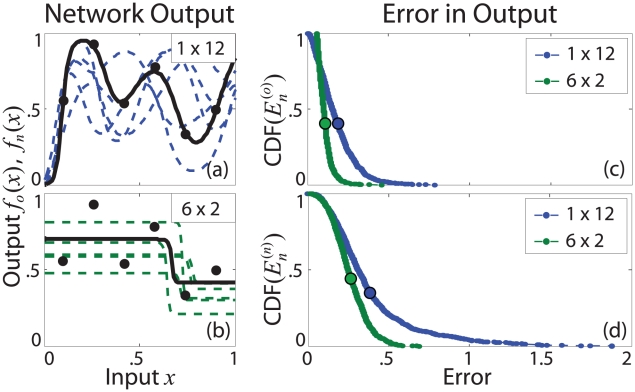
Network solutions and error distributions. Panels (a) and (b) show solutions produced respectively by the fan and stacked networks, indicating for each network the approximation 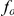 (solid curve) of the original points (point markers) and a subset of approximations 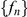 (dashed curves) of the new and buffer points. In this realization, the fan network fits the original points with a high order polynomial, while the stacked network produces a largely linear fit. Subsequent approximations 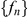 retain these features of 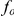. Panels (c) and (d) respectively show the CDFs of 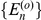 and 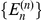, with the average value of each distribution marked by a filled circle. (c) The fan network achieves a lower minimum but higher maximum error on the original points than does the stacked network, resulting in a wider distribution with a higher average error. (d) Both networks produce low minimum errors on the new points, but the fan network again produces higher average and maximum errors than does the stacked network. These results are qualitatively similar given larger networks (Figure S1) and different sets of original points (Figure S3).

The network trains on six “original” points 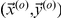 whose values remain fixed for all simulations. The original points are chosen to be evenly spaced in 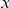 (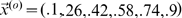) and random in 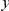 (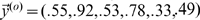). Similar behavior is observed for different choices, including permutations, of the specific values used here (see Figure S3). The original points represent the information we wish the network to remember during subsequent training. The network is given 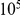 iterations to generate a functional representation 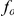 of 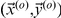 (see second panel of Figure 1(b) and Figures 2(a) and 2(b)), and training ceases if the error plateaus (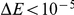 for 1000 iterations). We refer to this situation as allowing “unlimited” training time because in practice, the network finds a solution before reaching the maximum number of iterations.

### Second Training Session

#### Step 2.1 - Sample

The set of weights that produce 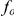 forms the starting point for the second training session. We refer to this state of the network as the “sampled state” in order to distinguish it from the randomly initialized state chosen prior to the first training session. In this state, the network randomly samples a pool of 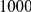 buffer points 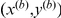 from 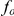 (see third panel of Figure 1(b)). This is accomplished by (*i*) randomly choosing input 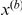 values from 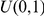 and (*ii*) computing the corresponding output 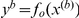 values using the set of network weights that produce 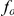. Subsets of buffer points, which lie along the functional representation 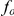 of the original points, are used in the following step to simulate memory rehearsal.

#### Step 2.2 - Re-train

The network re-trains on six new points 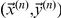 and six buffer points 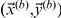 (see fourth panel of Figure 1(b)). New points are chosen by randomly selecting six independent 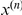 and 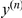 values from 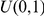. Buffer points are chosen by randomly selecting, with uniform probability, six 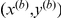 pairs from the pool of the buffer points generated in *Step 2.1*. Training on the same number of new and buffer points places equal emphasis on learning and memory rehearsal. Because the new points are randomly chosen and poorly constrained, we repeat the second training session 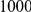 times to generate a distribution of solutions 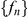 (see Figures 2(a) and 2(b)). Both the new and buffer points vary from session to session, but the buffer points are always sampled from the same original function 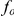. We restrict the training time of each session to 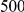 iterations, thereby giving the network “limited” time to learn.

#### Notation

We use the super and subscripts “
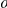
” and “
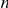
” to refer respectively to the “original” and “new” points, 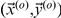 and 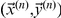, and functional approximations, 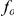 and 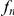. Each function 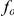 produces a single error value 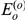 measured with respect to 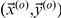. Each set of functions 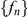 produces two sets of error values, 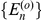 and 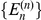, measured with respect to 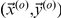 and 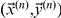, respectively.

## Results

### Tradeoffs in Learning and Memory Tasks

We train the five networks shown in Figure 1(a), first considering the differences between the boundary fan (parallel) and stacked (layered) networks. Given the large number of adjustable parameters 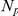 relative to the small number of training points 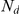, we expect all five networks to fit the points with high accuracy. Instead, the networks show significant differences in performance both within individual training sessions and measured statistically over many sessions. These results, discussed in detail below, show the same qualitative features for larger networks (Figures S1 and S2) and for different sets of original points (Figures S3 and S4).

#### Fan and stacked architectures

Examples of the solutions 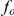 and 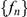 produced by the fan and stacked networks are shown in Figures 2(a) and 2(b). Each set 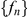 is characterized by errors 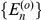 and 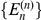, which measure the ability of the network to retain and learn information, respectively. The cumulative distribution functions (CDFs) of these errors are shown in Figures 2(c) and 2(d), where the CDF gives the probability that the network produces an error greater than 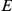 for any value of 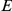.

The fan and stacked networks produce qualitatively different types of solutions 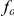 and 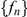. While the specific functional form of 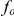 depends on the randomly initialized network state (see the following section), the 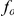 solutions shown here have errors that are representative of the average network performance over a range of randomly initialized states. The stacked solution 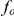 averages over the variation in the original points (Figure 2(b)). In contrast, the fan solution 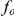 accurately fits all six original points with a high order polynomial (Figure 2(a)). In both networks, subsequent solutions 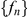 retain the features of 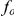. Because the sigmoid transfer function (see Methods) is identical for all nodes, the differences between the fan and stacked solutions arise solely from variations in network architecture. As the sigmoid function maps an infinite input space to a finite output space bounded between 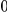 and 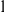, successive applications of sigmoids produced by serial (stacked) computations tend to result in linear or step function outputs, while a sum of sigmoids produced by parallel (fan) computations tends to result in highly variable outputs.

The interference between the two training sessions results in the deviation of 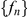 from 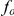, which tends to increase 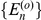 relative to 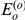. We find that in its best case, the stacked network shows no deviation in 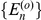 from 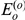. In contrast, the fan network shows a minimum deviation of 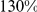 and a higher deviation on average compared to the stacked network. This deviation measures the ability of the network to retain the original representation 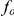, regardless of how erroneous that representation may be. Although the stacked network generates a higher error representation of the original points during the first training session, it can more accurately retain this representation when presented with new points.

The minimum and maximum values of 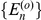 measure the best success and worst failure of the network in retaining old information while avoiding interference from new information. While the bounded output space limits the maximum error, linear solutions tend to further restrict these bounds. As a result, the stacked network has a lower maximum error at the cost of having a higher minimum error, as shown in Figure 2(c). In contrast, the fan network can retain the original information more accurately by achieving a lower minimum error, but it can also fail more catastrophically with a higher maximum error.

Similar features are observed in the distributions of 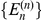 shown in Figure 2(d). The minimum and maximum values of 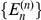 measure the best success and worst failure of the network in learning new information while attempting to retain old information. While both networks achieve low minimum error at their best, the fan network produces a much larger maximum error than does the stacked network. In addition to achieving more extreme best and worst cases, the fan network also has higher average error values 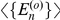 and 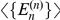.

#### Intermediate architectures: Tradeoffs in learning and memory

We extend this analysis to the intermediate architecftures shown in Figure 1(a), organizing the results based on the degree of network serialization 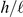 (a purely geometrical factor).

**Figure 3:**
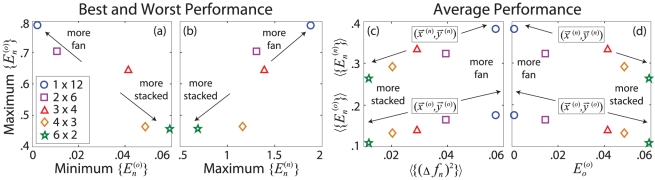
Tradeoffs in network learning and memory. Best, worst, and average network performance is measured with respect to solutions 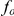 and 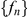 produced by the five networks shown in Figure 1(a). With respect to solutions 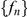 produced during the second training session, increasing 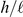 (a) decreases the maximum value of 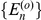 at the cost of increasing its minimum value, (b) decreases the maximum error in both 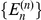 and 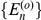, and (c) decreases the average solution variance 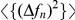 and the average errors 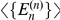 and 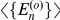. (d) Increasing 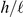 increases 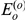 achieved during the first session but decreases 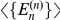 and 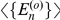 achieved during the second session. These results are qualitatively similar given larger networks (Figure S2) and different sets of original points (Figure S4).

Tradeoffs in performance are observed across the range of architectures. For example, in Figure 3(a), we see a tradeoff between the minimum and maximum values of 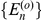. As 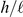 increases, the network does not fail as badly in its worst case but also does not succeed as well in its best case. Figure 3(b) shows that increasing 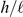 decreases the maximum error in both 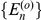 and 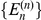, indicating that the stacked architecture is best suited for minimizing failure in both learning and memory. Figure 3(c) shows that increasing 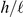 decreases both the average solution variance 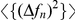 and the average errors 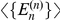 and 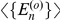. While we might naively expect that high solution variance (fan) would indicate a flexible network able to accurately fit nonlinear data, we instead find that high variance leads to high average error. In contrast, low variance, linear solutions (stacked) tend to minimize average error.

Furthermore, we find a tradeoff in performance between the first and second sessions, shown in Figure 3(d). Increasing 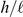 worsens performance during the first session by increasing 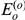 but improves average performance during the second session by decreasing both 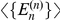 and 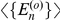, suggesting a tradeoff between the accuracy and generalizability of network solutions. The fan network, which produces a very accurate, specific representation of the original points, shows a much higher average error when it tries to generalize this representation. In contrast, the coarser representation produced by the stacked network is better able to incorporate new information.

### Adaptation to Variable Learning Conditions

Both natural and artificial systems can be found in a variety of states when presented with new information. The success in learning this information may depend both on the initial state of the system and on the learning conditions. We explore these possible dependencies by varying both the randomly initialized network state and the training conditions.

#### Variable initialized states

Because the conjugate gradient descent algorithm (see Methods) is deterministic, the randomly initialized state determines 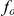, which then influences subsequent solutions 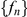.

To study the influence of random initialization on 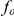, we train all five networks on the original points with 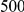 sets of randomly chosen weights, allowing “unlimited” training time. Each network produces a set of 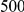 functions 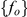 with error values 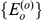.

**Figure 4:**
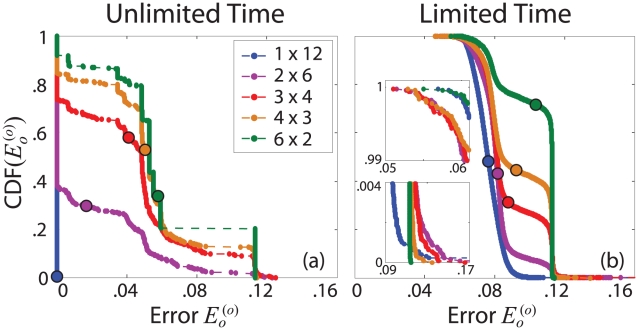
Network performance under variable learning conditions. CDFs of 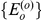 are shown given (a) unlimited and (b) limited training time for the five networks shown in Figure 1(a). (a) The fan network consistently finds zero error solutions, while all other networks find solutions with a range of error values. (b) Intermediate networks find lower error solutions than do the fan and stacked networks (upper inset). Increasing 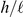 significantly decreases the both the maximum error and the frequency of high error solutions (lower inset). In both (a) and (b), increasing 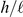 increases 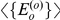 (filled circles).

The CDF of 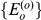, shown in Figure 4(a), reveals that the fan network consistently finds zero error solutions, while all other networks find solutions with a wide range of error values. The networks can collectively produce both zero error and high error solutions and do so with probabilities that respectively decrease and increase as 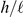 increases. The discontinuities in the stacked error distribution may indicate that the error landscape is composed of localized sets of minima with distinct depths. In comparison, the intermediate distributions show greater continuity in error, suggesting the presence of a larger number of connected minima with variable depths.

The distributions are more heavily weighted toward high error as 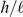 increases, thereby increasing the average error 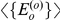. For a given architecture, the average number of training iterations decreases with increasing solution error, indicating an inherent tradeoff between speed and accuracy. While able to produce solutions with the same degree of accuracy as the fan network, the intermediate and stacked networks can also quickly produce coarse solutions. However, the intermediate networks require fewer iterations than the stacked network to reach solutions of similar error, suggesting that the presence of additional connections may facilitate faster performance.

If we inspect the solutions produced by each network, we find that low, medium, and high error solutions correspond respectively to fitting all, some, or none of the points with a high order polynomial and fitting the remaining points with a horizontal line. To emphasize differences in network performance, the solutions 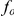 used to generate the results shown in Figures 2 and 3 were chosen because their error was representative of the distribution averages shown in Figure 4(a).

#### Temporal constraints

In natural systems, the time allowed to gather information from the environment is often limited, and a highly specific representation of information may not be desirable or even attainable. To investigate the effect of temporal constraints, we train the five networks on the original points with 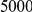 sets of randomly chosen weights, now terminating training after 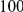 iterations. The increased number of randomly initialized states allows us to better resolve the edges of the error distributions shown in Figure 4(b).

Once training time is limited, all distributions shift toward higher error values, again revealing a tradeoff between speed and accuracy. As before, 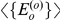 increases as 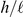 increases. Discontinuities in the distributions are also removed, indicating that the networks do not have sufficient time to consistently find distinct sets of minima.

The dynamic range of performance decreases as 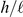 increases, resulting in significant differences between the edges of each distribution. At the rightmost edge, both the frequency of high error solutions and the maximum error value increase as 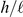 increases. The stacked network shows an abrupt cutoff near the minimum error achieved by fitting the original points with a horizontal line. All other distributions extend beyond this value. In contrast to the case of unlimited training time, the fan network shows the least consistency in performance and produces several catastrophic errors, thereby revealing the greatest sensitivity to changes in training time. At the leftmost edge of the distributions, the intermediate networks find lower minimum error values than do the fan and stacked networks. This is similar to the behavior observed with unlimited training time, where the intermediate networks found comparable solutions to the fan and stacked extremes in fewer iterations. It may therefore be interesting in the future to verify the dependence of performance on the number of network connections.

### Dependence on Error Landscape Structure

Given unlimited training time, the distributions in Figure 4(a) mark the error of local minima found within the error landscape of each network. Each minimum can be characterized by the degree of local landscape curvature, where directions of high curvature specify combinations of weight adjustments that produce large changes in error. We adopt the terminology used in previous studies and refer to directions with high and low curvature as stiff and sloppy, respectively [31], [32]. Stiff and sloppy directions are found by diagonalizing the error Hessian 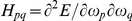 evaluated at the set of weights that produces the local error minimum. For computational efficiency, we use the approximate Levenberg-Marquardt (LM) Hessian [33], defined as:
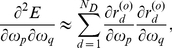
(2)where 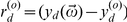 is the residual of the 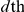 original point.

The LM Hessian is a good approximation to 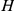 when the error of local minima, and thus the residual 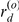, is small and the additional Hessian term 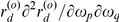 can be neglected. For a given model and data set, the LM Hessian agrees well with the stiffest eigenvectors of 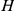 and is equivalent to 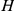 when the model perfectly fits the data. In addition, it has a known number of exactly zero eigenvalues equal to the difference in the number of model parameters 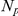 and the number of data points 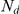 [31], [32].

We diagonalize the LM Hessian about each of the 500 minima with the error values 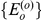 shown in Figure 4(a). Each error minimum produces a set of 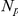 eigenvalues 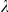 and normalized eigenvectors 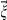, which give the degrees and directions of stiffness in weight space.

**Figure 5:**
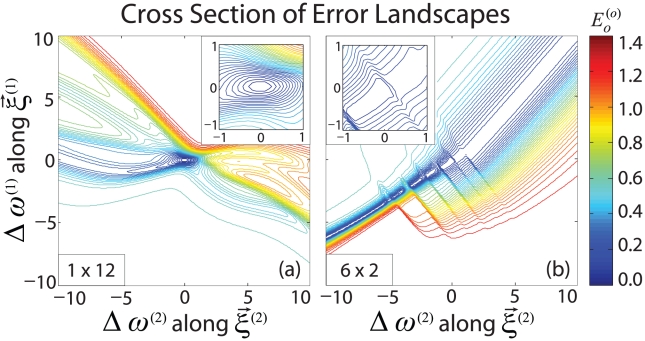
Network error landscapes. Error 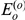 is projected onto the two stiffest eigenvector directions 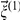 and 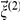 about minima produced by the (a) fan and (b) stacked network given unlimited training time. The two minima were chosen for comparison because they have the same number and similar magnitude of nonzero eigenvalues, although similar behavior was observed for alternative minima. The insets show zoomed in views of the contour plots about their central minima. (a) The projection of the fan landscape shows a single deep minimum surrounded by smooth peaks. (b) In contrast, the projection of the stacked landscape shows a long, deep valley of several local putative minima separated by low barriers. The surrounding landscape is much bumpier than that of the fan network.

As an illustrative example of landscape features observed along these relevant directions, Figures 5(a) and 5(b) show the projection of the error landscape onto the two stiffest eigenvector directions 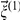 and 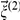 centered on zero error minima produced by the fan and stacked networks, respectively.

The fan landscape shows a single deep basin surrounded by smoothly varying peaks. In contrast, the stacked landscape is rugged, showing a deep valley with several minima separated by small barriers. While these minima appear to be distinct, they may be connected by higher dimensional pathways that cannot be seen in this reduced space.

#### Participation of network connections

The ability of a network to move along relevant eigenvector directions may depend on the number of weights that must be significantly adjusted, or equivalently the localization of eigenvector components. To quantify the degree of localization of the 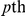 eigenvector 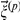, we calculate its participation ratio 
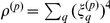
 [34], where individual eigenvector components 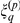 correspond to specific weights 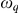 in the network. 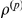 is a dimensionless quantity that ranges between a completely delocalized minimum of 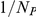, for which all components have equal weight 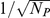, and a completely localized maximum of 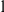, for which a single component carries unit weight.

**Figure 6:**
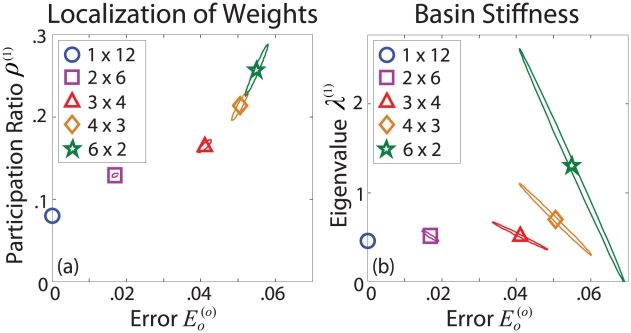
Properties of network error landscapes. Covariances between (a) 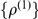 and 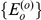 and between (b) 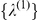 and 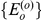 are shown for error landscape minima produced by the five networks shown in Figure 1(a). For each network, the values of 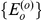 are taken from the distributions shown in Figure 4(a). Covariances, indicated by ellipses, are centered about their average values, indicated by markers. The semimajor axis of each ellipse marks the direction of maximum covariance. Increasing 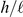 increases both the average and variance in all three quantities. For a given network, larger values of 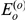 generally correspond to smaller values of 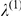 and larger values of 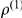.

For the set of minima with error values 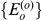, we quantify 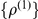 and 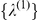 of the stiffest eigenvectors 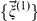, as combinations of weight changes specified by these eigenvector directions produce the largest changes in error. The covariances 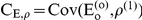 and 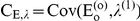 in these quantities are shown by the ellipses centered about their average values in Figures 6(a) and 6(b), respectively.

Figure 6 highlights the variability in basin structure within and between the networks. As 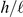 increases, both the average and variance in 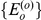, 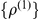, and 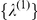 increase. Higher variance leads to lower confidence in predicting the success of the network, but it also suggests that the network has more options when exploring its error landscape.

The orientations of the covariance ellipses in Figures 6(a) and 6(b) provide information regarding the relationships between 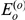, 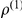, and 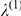. The semi-major axis of each 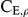 ellipse in Figure 6(a) lies along the trend swept out by the average values of 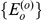 and 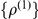, suggesting a general, positive correlation between 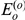 and 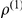. While the average values of 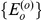 and 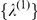 would suggest that these quantities are also positively correlated, Figure 6(b) shows that for a given value of 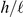, larger values of 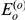 correspond to smaller values of 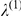. These results reveal general characteristics of error landscape structure; higher error minima (larger 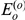) tend to be shallower (smaller 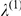) and require the adjustment of fewer weights (larger 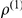).

#### Landscape characteristics and successful learning

Variations in landscape structure provide insight into the way in which each network searches for solutions. In particular, fan solutions are characterized by low error and participation ratio, indicating that the fan network must adjust nearly all of its weights in order to navigate zero error basins. In contrast, stacked solutions span a range of error values. The corresponding basins are characterized by a variety of eigenvalues and participation ratios, indicating that the stacked network can navigate many types of basins by adjusting variable numbers of weights. Larger participation ratios correspond to higher error and lower eigenvalues, suggesting that the stacked network can navigate shallow, high error basins by adjusting only a few of its connections. Narrow, low error basins, found by both the fan and stacked networks, require fine tuning of a larger number of connections.

In combination, landscape characteristics help explain the results shown in Figures 3 and 4. Given unlimited training time, landscape variability is disadvantageous and can prevent a network from finding a low error minimum. Once time is limited, landscape variability can be advantageous in preventing failure by providing the network with high error, shallow basins that can be navigated with the adjustment of relatively few connections. If limited training time is coupled with extremely noisy information, landscapes with high error basins can be advantageous by decreasing average error relative to landscapes with no easily reachable basins. Because our sequential sessions combined both limited and unlimited training time and both clean and noisy data, we see an additional tradeoff between the two sessions. Unlimited training time and well constrained data favor the fan over the stacked network in minimizing average error, while limited time and noisy data favor the stacked network over the fan.

## Discussion

In this study, we investigated the tradeoffs in learning and memory performance that arise from structural complexity. Importantly, none of the architectures considered here simultaneously mastered both learning and memory tasks, which suggests that systems whose function depends on such simultaneous success might require architectures that are complex combinations of both parallel and serial structures. Indeed, this inherent sensitivity of function to underlying architecture may help to explain the high degree of variability evident in architectural motifs of large-scale biological and technical systems. For instance, in natural neuronal networks, cortical connection patterns display a variety of architectural complexities at varying spatial scales. Examples of fan architectures are found in hub-and-spoke motifs, which form an important part of the small-world architecture [35]–[37], as well as in the decomposition of cortical network architectures into subnetworks or modules which may simultaneously process differential information [10], [38]–[41]. Moreover, stacked architectures are evident within cortical lamina [1], within the hierarchical organization displayed in the sequential ordering of the visual system [42], and within the nested modularity of large-scale cortical connectivity [10], [41], [43]. Similarly, artificial neural networks display complex combinations of fan and stacked motifs including modularity [44], hierarchy [45], and small-worldness [46], [47].

### Parallel versus Layered Architectures

Given the wealth of structural motifs present in real world systems, it is of interest to first isolate the tradeoffs in performance associated with small parallel and layered network structures which together form the complex architectural landscape of larger systems and thereby constrain their overall performance. Here we found that the deep, narrow basins within the error landscape enabled the fan network to produce very accurate solutions. However, the difficulty of simultaneously adjusting many network connections in order to escape deep basins may have hindered the ability of the fan network to adapt, a result that helps explain the susceptibility of parallel networks to the problems of overfitting and failure to generalize [26]. In contrast, higher variability in the width and depth of local minima enabled the stacked network to quickly find coarse but generalizable solutions through the adjustment of a smaller fraction of weights. In combination, these results support the hypothesis that the number and width of local landscape minima may increase with increasing number of hidden layers [4], and we suggest that this variability helps explain why layered networks may require fewer computational units and may better generalize than parallel networks [49], [50]. However, the impact of structural variations on functional tradeoffs, for example between specificity and generalizability, extends beyond artificial network studies and is crucial for understanding the interaction of learning processes in large scale models of the brain [51]. While parallel architectures are often preferred in artificial network studies due to their consistency and accuracy [48], [50], our results highlight the advantages of layered architectures when performance criteria favor generalizability and minimization of failure.

### Intermediate Architectures

Building on the intuition gained from the two benchmark extremes – fan and stacked – we further assessed the characteristics of intermediate networks, which can be used to more directly probe the expected behavior of structurally complex composite systems. In particular, our intermediate structures were composed of several adjacent stacked networks and therefore shared principal features of both parallel and layered systems. Additionally, these networks had slightly larger numbers of connections than the fan and stacked networks.

Due to these structural differences, the depth of local minima within the intermediate landscapes displayed more variation than fan minima but more continuity than stacked minima. As landscape variability was linked to improved generalization capabilities, a continuous range of basin depths may have enabled the more successful balance between flexible learning and stable memory observed in the intermediate networks. This performance supports the hypothesis that short path lengths (similar to the serialization 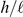 [52]) and low connection densities may facilitate simultaneous performance of information segregation (memory retention) and integration (generalization) within natural neuronal systems [53]. These competing processes are also maintained in natural neuronal systems and neural circuit models through homeostatic plasticity mechanisms such as synaptic scaling [54], [55] and redistribution [56], [57], in addition to the rehearsal methods employed here [19]–[23]. Even in the absence of such homeostatic plasticity mechanisms, we found that the architectural combination of parallel and layered connectivity helped foster a balance between learning and memory.

### Variable Learning Conditions and Network Efficiency

We extended our analysis from the case of unlimited training time, which revealed information about error landscape structure, to the biologically-motivated case of limited training time. Comparison of these two cases revealed a tradeoff in performance between training speed and solution accuracy. In the absence of temporal constraints, the production of highly accurate representations required longer training times. Similarly, temporal constraints led to larger solution errors. This tradeoff between speed and accuracy has been observed in cortical networks, where emphasis on performance speed during perceptual learning tasks increased the baseline activity but decreased the transient task-related activity of neurons within the decision-making regions of the human brain [58], [59]. Here we found that network architecture played a significant role in the manifestation of this tradeoff, and the presence of additional hidden layers helped minimize network susceptibility to changes in training time. In particular, the fan network demonstrated the greatest change in performance under temporal constraints, showing a decrease in consistency coupled with occasional catastrophic error values. In contrast, the intermediate and stacked networks improved consistency and minimized inaccuracy once training time was limited.

Upon closer inspection, we found that the intermediate networks produced solutions with increased speed given unlimited time and with increased potential for accuracy when time was limited as compared to the fan and stacked extremes. The presence of additional connections may have influenced the number of iterations required to find a solution, or similarly the minimum error found with a fixed number of iterations. While the graph measure of path length is known to influence network efficiency [52], these results imply that the number of networks connections may additionally enable the network to quickly find an accurate solution.

In addition to static variations in connectivity, dynamic structural changes such as synapse formation [60] can facilitate learning and memory processes. The converse case of network degradation, or disruptions to structural connectivity, is also known to have widespread consequences in functional properties of the brain [61]–[63]. A more detailed study of the relationfship between connection number and robustness could provide additional insight into the effects of synapse formation and degradation on functional performance. Our analysis of error landscape features revealed that different architectures showed variable localization properties in the eigenvectors associated with local error minima, and we therefore expect robustness to depend on both the architecture and the location of growth or damage within the network.

### Methodological Considerations

We found that parallel networks suffered from the creation of excessively detailed representations of information, an “overfitting” problem that is often addressed through the use of cross-validation [64] and weight regularization [65] techniques. As one goal of this study was to uncover the structural basis for differences in representational capabilities, it was crucial to understand network behavior in the absence of task-specific cross-validation schemes. Additionally, as the number of parameters was roughly constant across all network structures (and identical for the fan and stacked networks), we were able to draw comparisons across network architectures in the absence of additional weight regularization constraints.

While parallel network models have commonly been used in machine learning studies, multi-layer “deep” networks have recently gained interest due to their potential ability to compactly represent (using fewer computational units and parameters) highly variable functions [49], [50]. The “deep belief” framework has been successful for training large, multi-layered networks, and training methods often couple unsupervised, layer-wise (greedy) training with supervised fine-tuning [66]. Recent studies of deep belief networks found that classification performance improved with the addition of layers [48]. In addition, it was suggested that a reduction in the number of hidden layers would require an exponential increase in the number of hidden units in order to achieve similar network performance [50]. These results emphasize the capabilities of layered networks and provide an additional framework in which to explore structure-function tradeoffs.

Although biologically-motivated, the FFBP framework includes several simplifying assumptions that could be modified to include additional, realistic complexity. First, we assumed that only the connection weights, analogous to synaptic strengths, were variable. Real neurons also exhibit changes in intrinsic dynamics [67] that interact with network architecture to constrain functionality in the brain [68]. Accounting for such relationships could be particularly relevant, for example, in the study of neuron response profiles within different cortical layers [13]. Second, we assumed that signals passed between nodes had no temporal structure, analogous to representing steady state neuron firing rates. Temporally varying signals could be included to study the dependence of dynamic properties, such as synchronization [68]–[70] and signal propagation [71], on structural organization [72]. Lastly, we assumed feedforward connectivity. The addition of recurrent connections could be used to study the relationship between recurrent structure and oscillatory functions such as cortical sleep rhythms [73] and oscillation couplings relevant for associative learning and memory [74]. In each of these directions, we anticipate that underlying structural complexity will continue to impact performance through functional tradeoffs.

### Conclusion

In summary, different network architectures produce error landscapes with distinguishable characteristics, such as the height and width of local minima, which in turn determine performance features such as speed, accuracy, and adaptability. Inherent tradeoffs, observed across a range of architectures, arise as a consequence of the underlying error landscape structure. The presence of local landscape minima enable greater speed, more generalizable solutions, and minimization of catastrophic failure. However, these successes come at the cost of decreased accuracy. Understanding how both the landscape characteristics and the resulting performance features vary across a range of architectures is crucial for both understanding and guiding the design of more complex biological and technical systems.

## Supporting Information

Figure S1
**Network solutions and error distributions produced by larger networks.** Panels (a) and (b) show solutions produced respectively by larger versions of the fan (1×18) and stacked (9×2) networks, indicating for each network the approximation 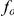 (solid curve) of the original points (point markers) and a subset of approximations 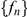 (dashed curves) of the new and buffer points. Panels (c) and (d) respectively show the CDFs of 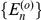 and 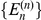. All results are qualitatively similar to those obtained using smaller networks (Figure 2).(EPS)

Figure S2
**Tradeoffs in network learning and memory observed in larger networks.** Best, worst, and average network performance is measured with respect to solutions 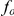 and 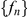 produced by networks of size 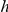
×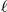 = 1×18, 2×9, 3×6, 6×3, 9×2. Panels (a) and (b) show the maximum values in 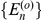 versus (a) the minimum values in 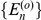 and (b) the maximum values in 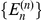. Panels (c) and (d) show the the average errors 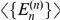 and 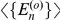 versus (c) the average solution variance 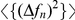 and (d) the original error 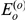. All results are qualitatively similar to those obtained using smaller networks (Figure 3).(EPS)

Figure S3
**Network solutions and error distributions produced using a permuted training function.** During the first training session, all networks were trained using the same random permutation of the original point values quoted in the main text. Panels (a) and (b) show solutions produced respectively by the fan and stacked networks, indicating for each network the approximation 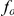 (solid curve) of the permuted set of original points (point markers) and a subset of approximations 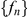 (dashed curves) of the new and buffer points. Panels (c) and (d) respectively show the CDFs of 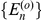 and 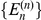. All results show the same qualitative features as those produced using the unpermuted set of original points (Figure 2).(EPS)

Figure S4
**Tradeoffs in network learning and memory observed with a permuted training function.** Best, worst, and average network performance is measured with respect to solutions 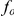 and 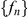, where 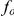 was generated using a random permutation of the original point values quoted in the main text. Panels (a) and (b) show the maximum values in 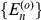 versus (a) the minimum values in 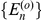 and (b) the maximum values in 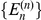. Panels (c) and (d) show the the average errors 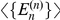 and 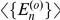 versus (c) the average solution variance 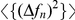 and (d) the original error 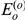. All results are qualitatively similar to those obtained using the unpermuted set of original points (Figure 3).(EPS)
